# Supplementary material for: Classifying cancer genome aberrations by their mutually exclusive effects on transcription
Source: BMC Med Genomics. 2017 Dec 21;10(Suppl 4):66. doi: 10.1186/s12920-017-0303-0 (PMC5763295; doi:10.1186/s12920-017-0303-0)
Supplement: Supplementary file 1 — Supplementary Figure S1- S7. (PDF 798 kb) [file 12920_2017_303_MOESM1_ESM.pdf]

Supplementary Material

Classifying cancer genome aberrations by their mutually exclusive effects on transcription

Jonathan B. Dayton, Stephen R. Piccolo

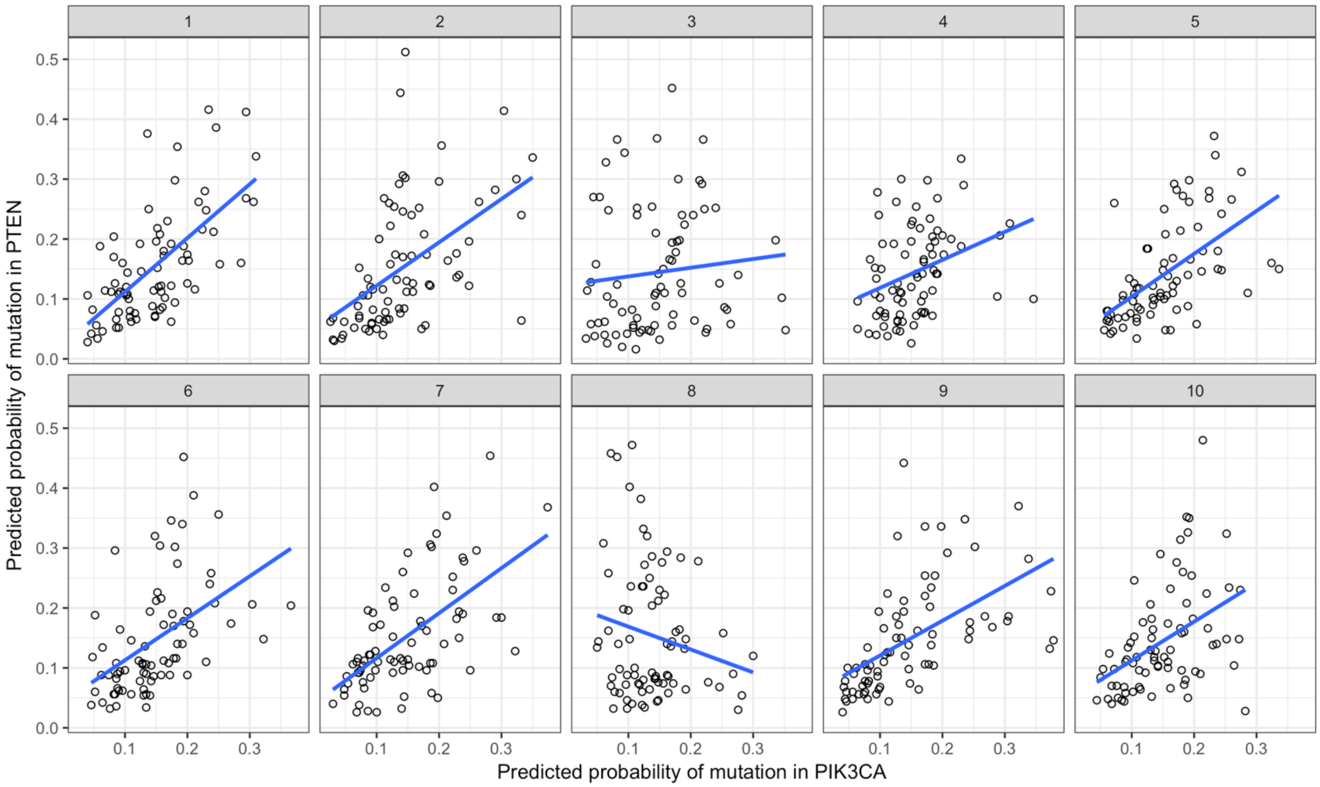

Figure S1: *Scatterplots depicting correlations between predicted probabilities for breast-carcinoma samples with a mutation in PIK3CA or PTEN*. Each scatterplot represents probabilistic predictions for a single iteration of cross validation.

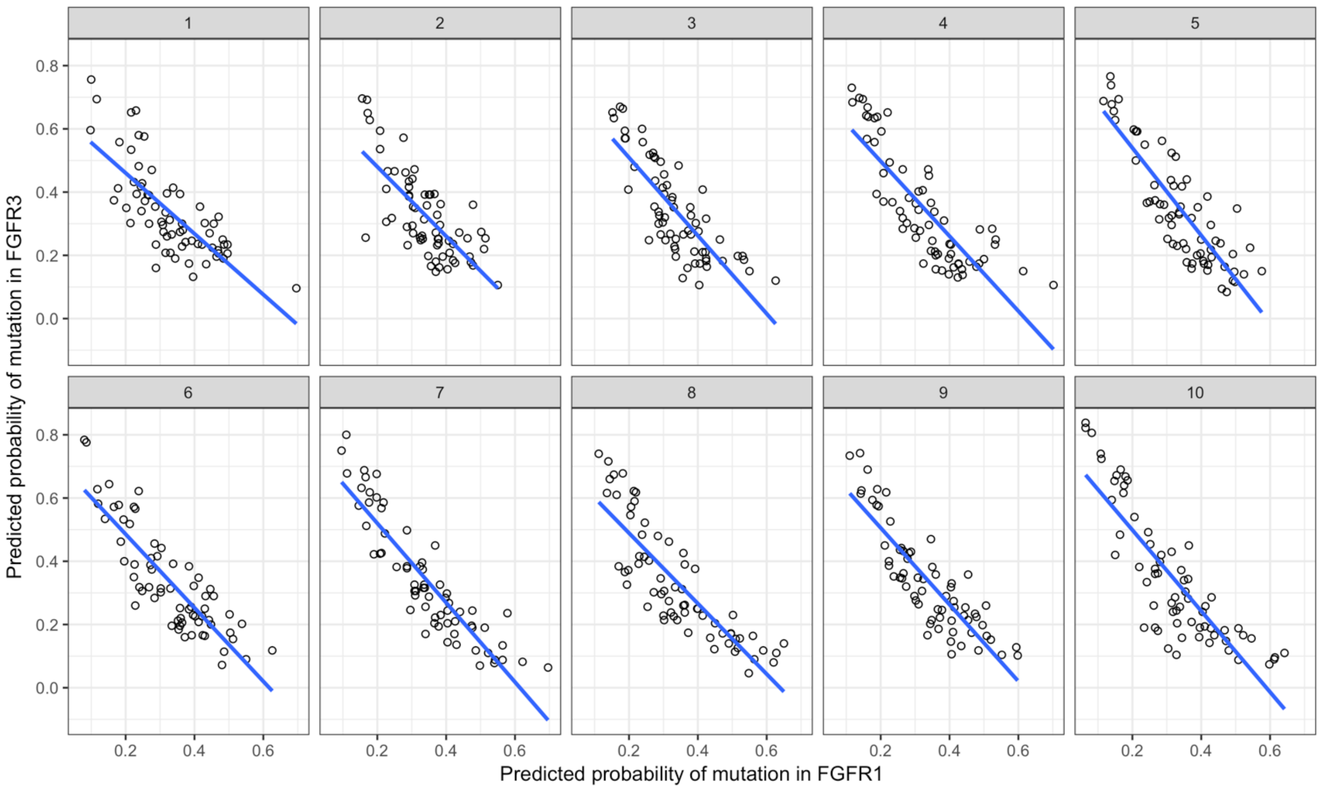

Figure S2: *Scatterplots depicting correlations between predicted probabilities for bladder-carcinoma samples with a mutation in PIK3CA or PTEN*. Each scatterplot represents probabilistic predictions for a single iteration of cross validation.

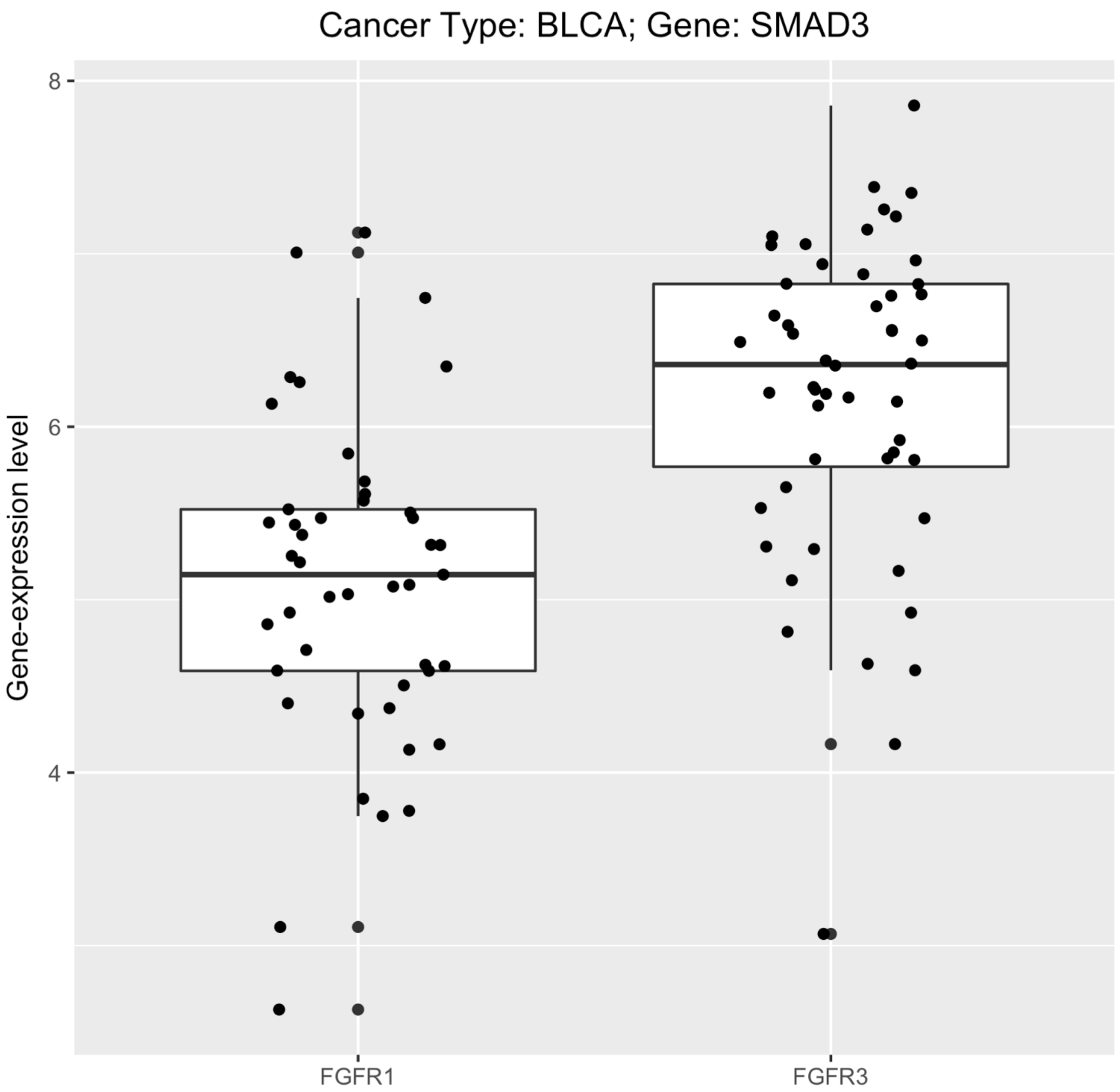

Figure S3: Expression levels of the *SMAD3* gene for bladder-carcinoma samples that possessed a somatic mutation in either FGFR1 or FGFR3.

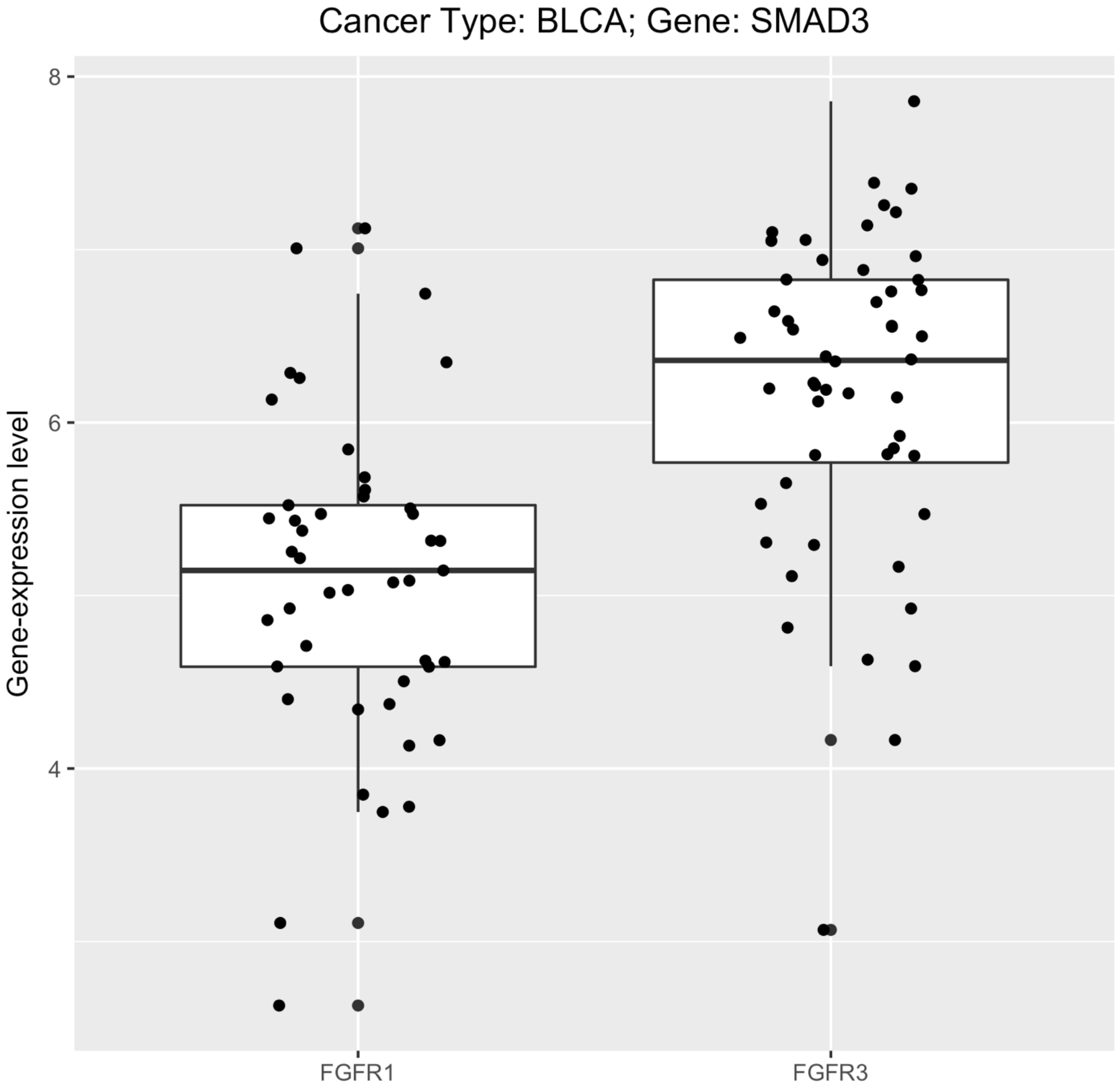

Figure S4: Expression levels of the *FGFR3* gene for bladder-carcinoma samples that possessed a somatic mutation in either FGFR1 or FGFR3.

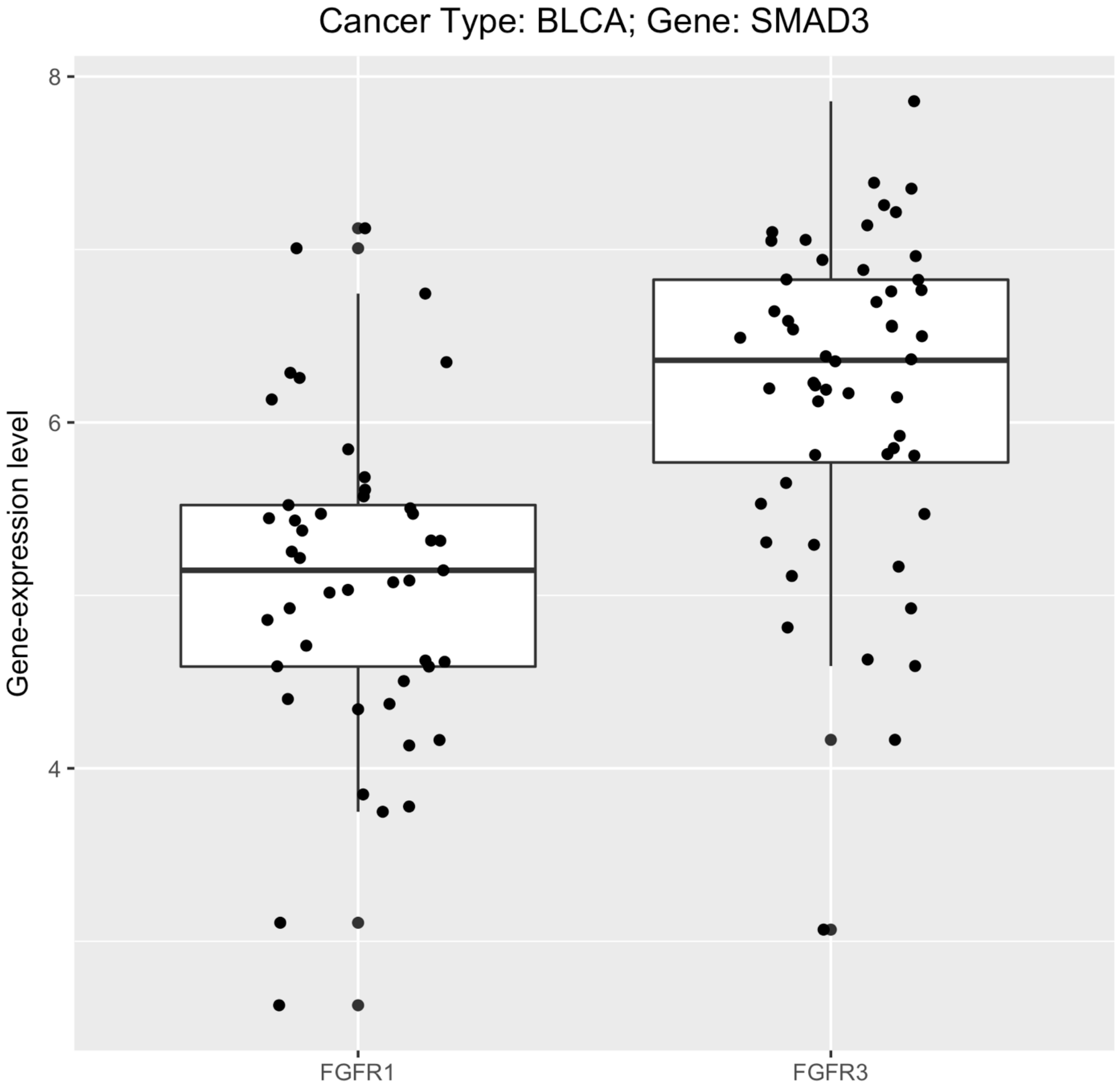

Figure S5: Expression levels of the *FN1* gene for bladder-carcinoma samples that possessed a somatic mutation in either FGFR1 or FGFR3.

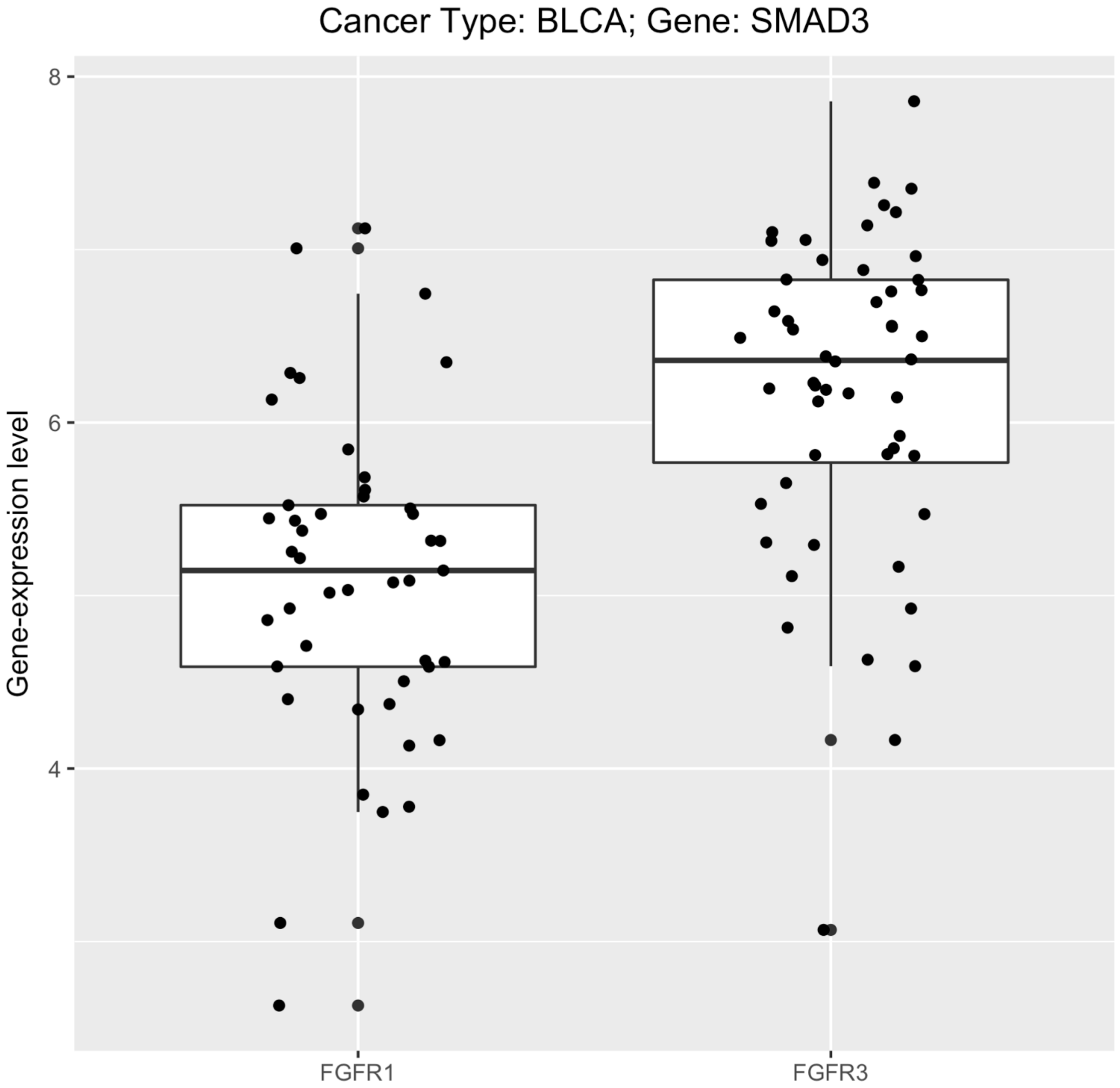

Figure S6: Expression levels of the *LAMA1* gene for bladder-carcinoma samples that possessed a somatic mutation in either FGFR1 or FGFR3.

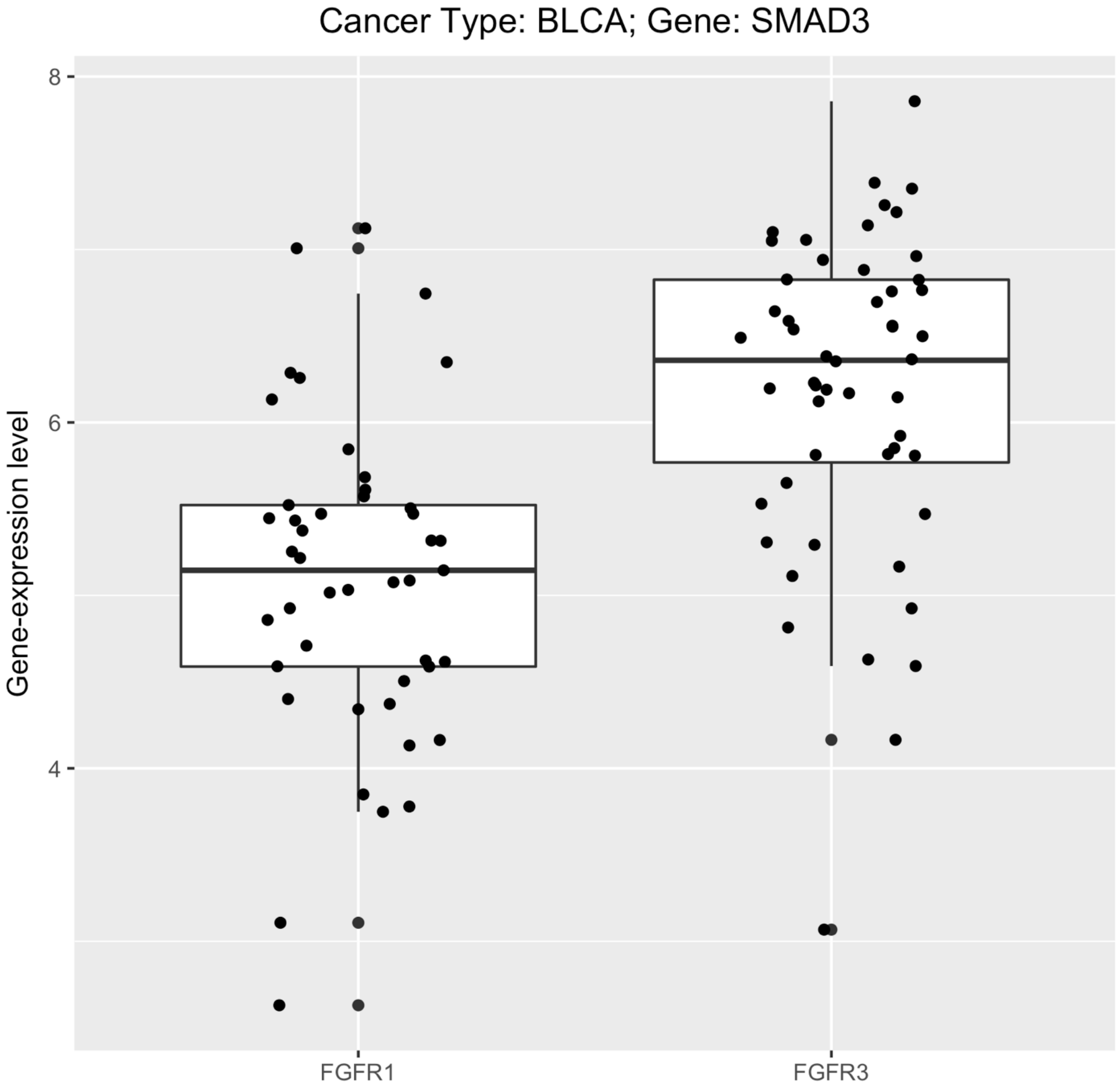

Figure S7: Expression levels of the *FGFR1* gene for bladder-carcinoma samples that possessed a somatic mutation in either FGFR1 or FGFR3.
